# Role of intergenerational connections in cognitive aging: Evidence from a Chinese longitudinal study

**DOI:** 10.3389/fpubh.2024.1396620

**Published:** 2024-08-21

**Authors:** Shanshan Hu, Jingjing Ge, Minglei Fang, Jingjing Yang

**Affiliations:** ^1^School of Public Health, Chongqing Medical University, Chongqing, China; ^2^Research Center for Medicine and Social Development, Chongqing Medical University, Chongqing, China; ^3^College of Humanities and Management, Guizhou University of Traditional Chinese Medicine, Guiyang, Guizhou, China

**Keywords:** CHARLS, cross-lagged panel model, intergenerational support, intergenerational connection, cognitive function, longitudinal research, middle-aged and older adult, rural China

## Abstract

**Objective:**

To explore the impact of intergenerational connections on cognitive function in middle-aged and older adults (45–60 years and over 60 years, respectively) and analyze the urban–rural and sex differences in the effects of intergenerational connections on cognitive function.

**Method:**

Based on China Health and Retirement Longitudinal Study data (CHARLS), this study conducted ID matching for four waves of data from 2011, 2013, 2015, and 2018. Cognitive function was measured via Telephone Interview for Cognitive Status-modified (TICS-m), word recall, and imitation drawing. Using a combination of cross-sectional and longitudinal research, we constructed the cross-lagged panel model (CLPM) with a sample of 1,480 participants to explore the relationship between intergenerational connections and cognitive function.

**Results:**

This study examines the impact of intergenerational connections on cognitive function in middle-aged (45–60 years) and older adults (over 60 years) using data from the CHARLS. It identifies urban–rural and sex differences, with notable effects among rural female participants. The frequency of meeting with one child negatively predicts cognitive function (*β* = −0.040, *p* = 0.041), and the frequency of communication with one child positively predicts cognitive function (*β* = 0.102, 0.068, 0.041, *p* < 0.001, *p* = 0.001, 0.045). Meanwhile, intergenerational connections with multiple children positively predicts cognitive function (*β* = 0.044, *p* = 0.031), (*β* = 0.128, 0.084, and 0.056, *p* < 0.001, 0.001, *p* = 0.008). There are urban–rural and sex differences in the effects of intergenerational connections on cognitive function; additionally, the effects of intergenerational connections on cognitive function are significant in rural female middle-aged and older adults.

**Discussion:**

This study proposes the theory of skewed intergenerational support, which suggests that as middle-aged and older adults age, the responsibility for intergenerational support is skewed toward one child. This leads to conflicts between middle-aged and older parents and the child, which further affects cognitive function. In addition, this study put forward the boat-carrying theory of intergenerational relations and “to hold a bowl of water level” is the art of dealing with intergenerational relationships.

## Introduction

In the wake of profound demographic shifts in China, the 21st century has witnessed the emergence of population aging as a critical social challenge (1). Recent data from the seventh national population census reveals a striking statistic: in 2020, the cohort aged 60 and above reached 264 million, representing 18.7% of China’s total population (2). This demographic trend poses formidable hurdles for the sustainable development of socio-economics and healthcare systems (3). Cognitive dysfunction stands out as a significant concern, notably impacting the physical and mental wellbeing of China’s middle-aged (45–60 years) and older (over 60 years) populations (4). Cognitive dysfunction, characterized chiefly by memory impairment, along with disturbances in attention, executive function, and visuospatial abilities (5), critically undermines the autonomy and life quality of these individuals. Its progression into severe stages necessitates long-term care, thus profoundly affecting the healthy life expectancy of this demographic (6, 7). Understanding the dynamics influencing cognitive function is therefore a vital step toward enhancing the cognitive health and overall wellbeing of middle-aged and older adults. This study aims to delve into these dynamics, offering insights that could inform strategies to bolster cognitive function in this increasingly significant segment of the population.

Convoy model of social relations posits that the network of family, friends, and other social connections plays a pivotal role in safeguarding an individual’s physical and mental health throughout their lifespan. This model underscores the value of social support and emotional satisfaction derived from these networks in fostering personal health and subjective well-being (8, 9). Meanwhile, the theory of cognitive reserve, suggests that variations in cognitive task processing leads to differential resilience against brain pathology or age-related changes (10). Research aligned with this theory has indicated that a healthy lifestyle and cognitively stimulating activities, such as engaging in leisure activities and social interactions, beneficially influence the cognitive abilities of older adults (11, 12). Furthermore, the theory of family modernization emphasizes the enduring importance of intergenerational interactions in providing daily care and emotional and economic support, even amidst societal modernization. The family unit remains a primary source of these support systems, with parent–child communication forming a crucial component (13). Based on these theoretical foundations, this study hypothesizes that the intergenerational connections between middle-aged and older adults and their children significantly impacts cognitive function. But more research needed on how visits from children impact middle aged and older adults cognitive health and should consider the heterogeneity of the form of intergenerational interactions. Therefore, even there is research suggest that intergenerational contact improves older people’s math test performance (14), understanding the different forms of intergenerational contact and the variations within and between families is crucial. Additionally, most studies focus on short-term effects, and long-term effects need further exploration. Our investigation seeks to explore this relationship, contributing to the nuanced understanding of cognitive health determinants in an aging population.

In the context of China’s urban–rural dual structure, significant health disparities shaped by long-standing differences in living environments and lifestyles have become increasingly evident. These disparities are particularly pronounced in the health outcomes of middle-aged and older adults across urban and rural areas, reflecting a complex interplay of socio-economic and environmental factors (15). The advent and proliferation of modern communication tools have also transformed the nature and frequency of interactions between these adults and their children, potentially leading to differential impacts on cognitive function based on geographic location. This study posits that the urban–rural dichotomy in China may extend to the realm of cognitive health, influencing the efficacy of intergenerational connections on cognitive function among middle-aged and older adults. Further complicating this dynamic are the documented sex differences in cognitive function development and decline in these age groups (16, 17). Given the variances in parent–child relationships between mothers and fathers, our research aims to shed light on how these sex differences might modulate the influence of intergenerational connections on cognitive health. By delving into these multifaceted relationships, this investigation seeks to contribute to a deeper understanding of the nuanced factors affecting cognitive function in China’s aging population, exploring the entwined influences of urban–rural settings and sex. Previous research has underscored the beneficial role of emotional connections with children in sustaining and enhancing cognitive function among middle-aged and older adults (18). However, the scope of existing studies is limited, with a predominant focus on rural populations and a tendency to utilize cross-sectional methodologies. This approach often overlooks the experiences of urban middle-aged and older adults and generally relies on subjective perceptions of familial relationships. Additionally, the methodological reliance on aggregating data across multiple children masks the nuanced dynamics of individual intergenerational interactions. Addressing these gaps, our study employs an innovative mixed-method approach, integrating both cross-sectional and longitudinal data. This approach allows for a more comprehensive exploration of the effects of intergenerational connections on cognitive function, incorporating objective measures of interaction frequency. Particularly pertinent in the context of China’s pension system, where typically one child assumes the primary caregiving role, our analysis distinguishes between the overall average of intergenerational interactions and the specific influence of the closest child–parent relationship on cognitive health. Furthermore, this study aims to illuminate the understudied aspects of urban–rural and sex-based differences in cognitive function. By providing a more holistic understanding of these dynamics, the study seeks to contribute valuable insights into the complex interplay of familial, geographical, and sex factors affecting cognitive health in China’s aging population.

Grounded in the preceding theoretical and empirical considerations, this study articulates a set of hypotheses aimed at unraveling the complex dynamics of intergenerational connections and cognitive function among middle-aged and older adults in China:

*H1*: For middle-aged and older adults, higher intergenerational meeting frequency with the closest child negatively predicts cognitive function. This hypothesis challenges the conventional notion of the benefits of frequent interactions, proposing that excessive contact might have diminishing returns or even adverse effects on cognitive health. Because in Chinese culture, intimacy but also a certain distance is the best, and it is important to balance this scale, because “too much is as bad as too little”.

*H2*: For middle-aged and older adults, higher intergenerational communication frequency with the closest child positively predicts cognitive function. This hypothesis underscores the potential cognitive benefits derived from regular, albeit perhaps less intensive, interactions with one’s children.

*H3*: The average level of intergenerational connections with multiple children has a positive correlation with cognitive function. This hypothesis reflects the cumulative benefits of diversified family interactions, suggesting that a broader network of familial support can enhance cognitive health.

*H4*: The impact of intergenerational connections on cognitive function is more pronounced among rural female middle-aged and older adults. This hypothesis is informed by the unique socio-cultural and intergenerational dynamics in rural China, where intergenerational relationships might play a more vital role in influencing cognitive health in this population due to greater reliance on children for old-age care and women’s closer relationships with adult children than men’s.

These hypotheses aim to deepen our understanding of the nuanced ways in which family dynamics and socio-cultural contexts influence cognitive function in China’s aging population.

## Methods

### Sources

**Figure 1:**
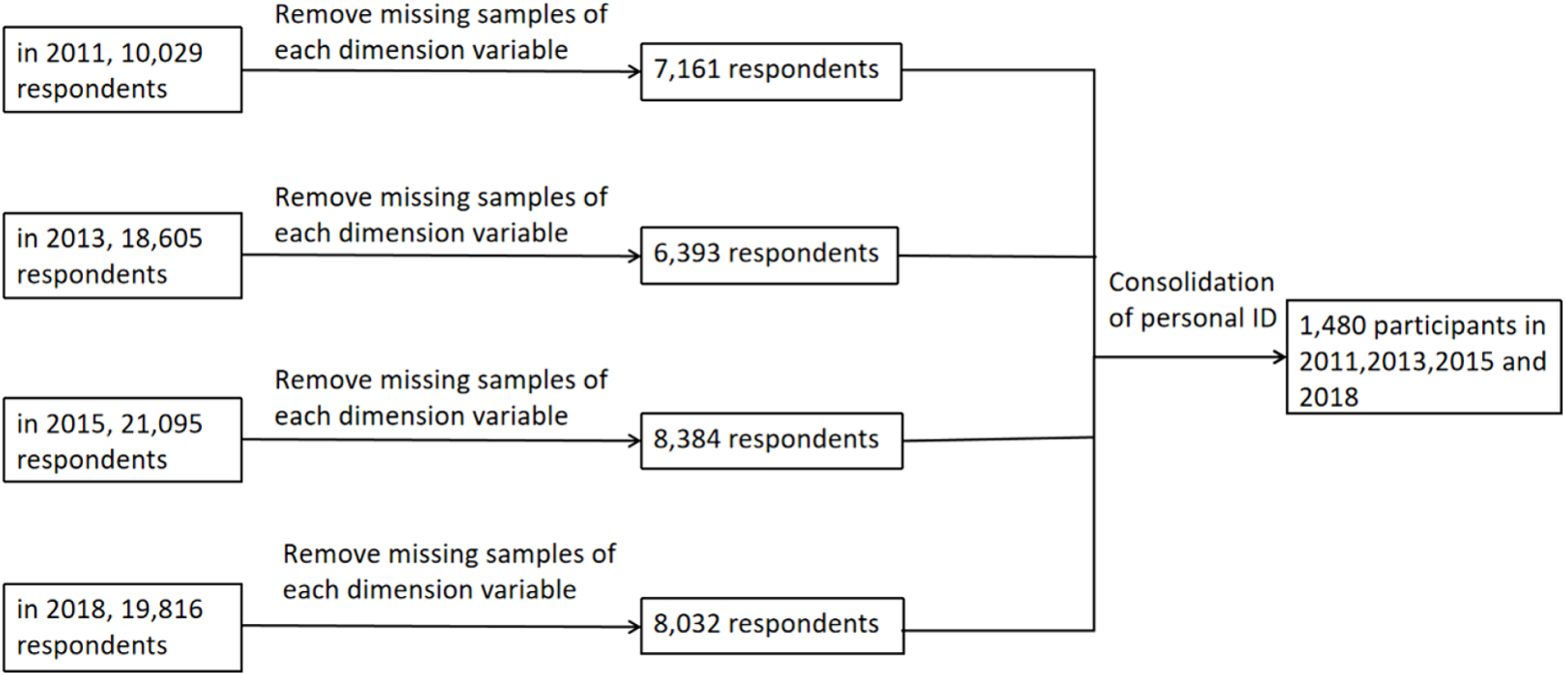
Data screening process.

This study utilizes data extracted from the China Health and Retirement Longitudinal Study (CHARLS) conducted in 2011, 2013, 2015, and 2018. The China Health and Retirement Longitudinal Study (CHARLS) is a comprehensive and representative database of middle-aged and older adults in China. Since 2011, CHARLS has been collecting data every 2 years from a wide-ranging sample across 150 counties and 450 communities (villages) in 28 provinces, by use of multistage stratified probability-proportionate-to-size sampling (19). By 2018, the study had surveyed approximately 19,000 respondents from 12,400 households (20). CHARLS is widely recognized for its high-quality data collection and management processes. It employs rigorous quality control measures, including professional auditors and verification processes conducted by the Chinese Center for Disease Control and Prevention. This ensures the reliability of the data, making CHARLS one of the most robust databases available for studying aging in China. The CHARLS dataset aligns well with our research objectives, which focus on exploring the relationship between intergenerational connections and cognitive function among middle-aged and older adults. CHARLS includes detailed measures on intergenerational support, cognitive assessments, and comprehensive demographic and family information. The quality and applicability of CHARLS data have been demonstrated in numerous high-impact publications (19, 21, 22), especially the paper about the cognitive aspects (23). Data collection in CHARLS was executed through standardized questionnaires during face-to-face interviews. These interviews were facilitated using computer-assisted personal interview techniques to ensure data accuracy and consistency. Prior to the commencement of data collection, all respondents provided written informed consent, adhering to ethical research standards (20). The IDs (individualID, householdID and communityID) used by CHARLS can be matched with there counterparts in the baseline sample. The community IDs are the same, but the household IDs changed from an 9 digit number to an 10 digit number in other waves, the 10th digit is an indicator for splitting household due to divorce. As the householdID changed in the other waves, the inidividualID also changed. In Stata, we can use the command “replace householdID = householdID + “0”; replace individualID = householdID + substr (individualID, −2,2)” to adjust the IDs in the baseline sample. For this study, the dataset was meticulously filtered to correct for abnormal and missing values and to match individual IDs, ultimately yielding a sample size of 1,480 middle-aged and older adults. The inclusion criteria were participants aged 45 years and above with complete data on meeting frequency and communication frequency, as well as cognitive function data across the four waves of CHARLS in 2011, 2013, 2015, and 2018. The exclusion criteria included (1) age below 45 years; (2) missing data on meeting and communication frequencies; (3) incomplete cognitive function data across the mentioned periods. The sample size in 2011 was 10,029. We excluded 2,868 individuals due to the exclusion criteria. The sample size in 2013 was 18,605. We excluded 12,212 individuals due to the exclusion criteria. The sample size in 2015 was 21,095. We excluded 12,711 individuals due to the exclusion criteria. The sample size in 2018 was 19,816. We excluded 11,784 individuals due to the exclusion criteria. And we matched the ID of 4 waves, which resulted in 1480 eligible individuals. A detailed data screening process is illustrated in Figure 1.

### Research methodology

#### Independent variables

In this study, intergenerational connections were categorized into meeting frequency and communication frequency. In CHARLS, meeting frequency was obtained by asking participants, “How often do you see (child’s name)?” and communication frequency was obtained by asking participants, “How often do you have contact with (child’s name) either by phone, text message, mail, or email, when you did not live with (child’s name)?” Detailed information such as the assigned values are shown in Table 1.

**Table 1:** Variables setting and assignment.

Variable settings	Assignment and range of values
Independent variables: intergenerational connections	
Meeting frequency	1 = other; 2 = almost never; 3 = once a year; 4 = once every 6 months; 5 = once every 3 months; 6 = once a month; 7 = every 2 weeks; 8 = once a week; 9 = 2–3 times a week; 10 = almost every day
Communication frequency	1 = other; 2 = almost never; 3 = once a year; 4 = once every 6 months; 5 = once every 3 months; 6 = once a month; 7 = every 2 weeks; 8 = once a week; 9 = 2–3 times a week; 10 = almost every day
Dependent variables:	
Cognitive function	0 ~ 21
Control variables:	
Sex	0 = female, 1 = male
Age (range)	45 ~ 108
Residence	0 = rural, 1 = urban
Wage income	0 = no wage income, 1 = wage income
Marriage status	0 = other, 1 = married and living with a spouse
Education level	0 = primary school education or below, 1 = primary school education or above
Chronic disease condition	0 = no chronic disease, 1 = suffering from chronic disease
Depression	0 ~ 30

Others refer to being unmarried, separated, divorced, or widowed.

The current study delineates intergenerational connections through two distinct lenses: meeting frequency and communication frequency. These variables are integral to understanding the dynamics of familial interactions in the context of cognitive function among middle-aged and older adults. In the CHARLS survey, the frequency of meetings with children was ascertained by posing the question, “How often do you see [child’s name]?” to the participants. This inquiry aims to capture the physical, face-to-face interactions between the participants and their children. On the other hand, communication frequency was gauged through the question, “How often did you have contact with [child’s name] either by phone, text message, mail, or email, when you did not live with [child’s name]?” This question is designed to encompass a broader range of interaction mediums, accounting for the various forms of remote communication that have become increasingly prevalent. The operationalization of these variables is meticulously detailed, assigning quantifiable values to the responses, as outlined in Table 1. This approach facilitates a nuanced analysis of the impact of these forms of intergenerational connections on cognitive function.

#### Dependent variable

The assessment of cognitive function in this study was meticulously conducted through telephone interviews, employing a comprehensive approach that included the Telephone Interview for Cognitive Status-modified (TICS-m), word recall, and imitation drawing tasks. These methods were strategically chosen to evaluate various cognitive dimensions such as orientation, arithmetic abilities, memory recall, and visuospatial skills, which are critical in understanding the cognitive health of middle-aged and older adults. The orientation component involved queries about the current year, date, season, and day of the week, aiming to assess temporal awareness. Arithmetic abilities were examined through a task involving serial subtraction of 7 from 100. The imitation drawing task, involving the depiction of two overlapping pentagons, was used to gauge visuospatial capabilities. Additionally, the cognitive evaluation included a delayed recall test involving 10 words to assess memory function. Responses were quantitatively scored, with each correct answer receiving 1 point. This scoring system produced a cognitive function score ranging from 0 to 21 (Cronbach’s alpha = 0.8), where higher scores are indicative of superior cognitive abilities. This scale, employed in our study, is a widely recognized and validated measure for cognitive screening (24), offering robust and reliable insights into the cognitive status of the participants.

#### Control variables

Variables setting and assignment are presented in Table 1.

### Statistical methods

This study utilized STATA17.0 to screen, match, and assign data to the CHARLS database samples from 2011, 2013, 2015, and 2018. The matched 1,480 nationally representative samples were used to statistically describe the data using mean and standard deviation (M ± SD) and rate (%). The 1,480 samples were tested using *t*-tests or ANOVA through SPSS27.0 to compare the four-year samples in terms of wage income, marital status, suffering from chronic diseases, depression, cognitive function, and intergenerational connections. STATA17.0 was used to conduct cross-sectional correlation regression analysis on the 4 time points samples (2011, 2013, 2015, and 2018), followed by longitudinal correlation analysis on the 1,480 samples. Due to the rich amount of cross-sectional data, it is possible to test the correlation of the samples with a large cross-sectional sample and use the 8 years of longitudinal tracking to reveal the causality of intergenerational linkage and cognitive function. The synthesis of cross-sectional and longitudinal study design better ensures the robustness of the study results. The cross-lagged panel model (CLPM) was constructed using Mplus 8.0 with 1,480 samples to explore the relationship between intergenerational connections and cognitive function. The differences between urban and rural areas and sex are explored. CLPM is a longitudinal statistical technique used to explore the dynamic relationship between variables. The CLPM reflects the interrelationships between variables through the correlation coefficient of cross-lagged paths, constructing the path of a variable’s prior level on the current level of that variable (called an autoregressive effect) as well as the path of its effect on the current level of another variable (called a cross-lagged effect). In longitudinal studies, the current value of the outcome variable as well as the predictor variable is affected by the prior level, and the CLPM can be sufficient to control for the effect of the prior level of the outcome variable (25, 26). The cross-lagged panel model is a well-established method for exploring causal relationships in longitudinal data. This method helps to address potential bidirectional effects and provides a clearer understanding of how changes in intergenerational connections influence cognitive function and vice versa (27). The validity of CLPMs in analyzing longitudinal data has been supported by extensive research (28).

## Results

### Descriptive statistics

Descriptive statistics of the variables were conducted (see Supplementary Table S1). There were 1,480 people, of whom 713 (48.2%) were female and 767 (51.8%) were male; the mean age ranged from 62.56 to 69.53 years old; 1,063–1,224 people (71.8–83.0%) resided in rural areas, and 251–417 (17.0–28.2%) resided in urban; 795–921 (53.7–62.2%) were married and living with a spouse, and 559–685 (37.8–46.3%) were not; 110–370 (19.1–25.0%) had a primary or above education level, and 467–1,123 (75.0–80.9%) had less than primary education level; 675–1,203 (72.2–85.4%) suffered from chronic diseases, and 173–408 (14.6–27.8%) did not; the maximum mean value of depression was 9.69, and the minimum mean value was 8.80. Middle-aged and older adults’ wage income, marital status, chronic disease status, and depression were statistically significant (*p* < 0.01), with an upward trend in having wage income and suffering from chronic disease and depression, and a downward trend in being married and living with a spouse.

The maximum mean value of cognitive function was 10.09, and the minimum mean value was 6.31. The difference was statistically significant (*F* = 319.880, *p* < 0.001), with a decreasing trend in cognitive function.

The maximum mean of the frequency of meeting with the closest child was 7.13, and the minimum mean was 6.78. The difference was statistically significant (*F* = 6.558, *p* < 0.001), and the meeting frequency with the closest child showed an increasing trend. The maximum mean of the communication frequency with the closest child was 7.24, and the minimum mean was 7.05 (*F* = 2.175, *p =* 0.089). The change in the frequency of communication with the closest child was more stable. The maximum mean value of the frequency of meeting with multiple children was 5.52, and the minimum mean value was 5.26. The difference was statistically significant (*F* = 5.012, *p =* 0.002), and the mean value of the frequency of meeting with multiple children showed a decreasing trend. The maximum mean value of the frequency of communication with multiple children was 6.57, and the minimum mean value was 6.11. The difference was statistically significant (*F* = 10.687, *p* < 0.001), and the mean of communication frequency with multiple children showed an increasing trend.

### Intergenerational connections with the closest child and cognitive function cross-sectional correlation regression between intergenerational connections with the closest child and cognitive function

Cross-sectional correlation analyses of intergenerational connections and cognitive function in 2011, 2013, 2015, and 2018 were conducted (Table 2). The correlation coefficient of meeting frequency and cognitive function in 2011 was −0.017, which was not significantly correlated. The correlation coefficients of meeting frequency and cognitive function in 2013, 2015, and 2018 were −0.042, −0.064, and −0.038, respectively, and all of them were significant at 1‰ of the significant level, showing a significant negative correlation. The correlation coefficients of communication frequency and cognitive function in 2011, 2013, 2015, and 2018 are 0.212, 0.211, 0.225, and 0.197, respectively, and all of them are significant at the 1‰ level of significance, showing a significant positive correlation.

**Table 2:** Results of the correlation regression analysis of the cross-sectional association between intergenerational connections with the closest child and cognitive function.

Variables	Correlation coefficient	Regression coefficient
MeetingT1 – CognitiveT1 (*n* = 7168)	−0.017	−0.026
CommunicationT1 – CognitiveT1 (*n* = 7162)	0.212***	0.34***
MeetingT2 – CognitiveT2 (*n* = 7951)	−0.042***	−0.068***
CommunicationT2 – CognitiveT2 (*n* = 6398)	0.211***	0.396***
MeetingT3 – CognitiveT3 (*n* = 10027)	−0.064***	−0.106***
CommunicationT3 – CognitiveT3 (*n* = 8397)	0.225***	0.439***
MeetingT4 – CognitiveT4 (*n* = 9546)	−0.038***	−0.047***
CommunicationT4 – CognitiveT4 (*n* = 8032)	0.197***	0.286***

T1 represents the baseline, and T2, T3, and T4 represent the follow-up visits. ***p < 0.001.

Cross-sectional regression analyses of intergenerational connections and cognitive function in 2011, 2013, 2015, and 2018 were conducted, and the results are shown in Table 2. The results showed that the frequency of meetings in 2013, 2015, and 2018 was a negative predictor of cognitive function, and the frequency of communication in 2011, 2013, 2015, and 2018 was a positive predictor of cognitive function.

### Longitudinal correlations between intergenerational connections with the closest child and cognitive function

The longitudinal correlation between intergenerational connections and cognitive function in 2011, 2013, 2015, and 2018 was analyzed (see Supplementary Table S2). The meeting frequency in 2011 and 2018 was significantly negatively correlated with cognitive function in 2013 and 2015, and not significantly correlated in 2011 and 2018. The frequency of meetings in 2013 and 2015 was significantly negatively correlated with cognitive function in 2015, and not significantly correlated at the other time points. The frequency of communication in the 4 time points has a significant positive correlation with cognitive function in all 4 time points.

### Cross-lagged panel analysis between intergenerational connections with the closest child and cognitive function

**Figure 2:**
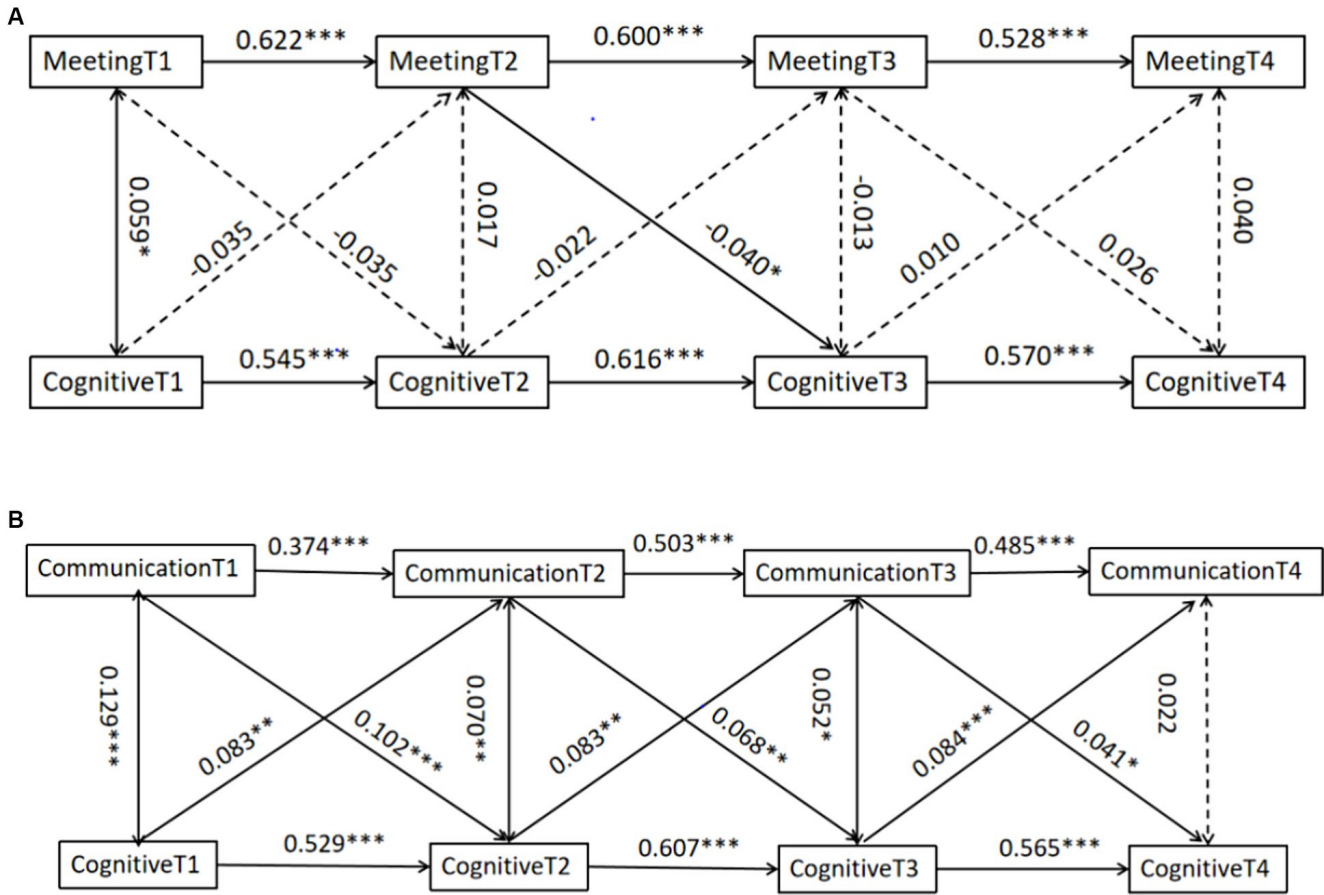
Cross-lagged panel model of intergenerational connections with the closest child and cognitive function. **(A)** Cross-lagged panel model of meeting frequency with the closest child and cognitive function. **(B)** Cross-lagged panel model of communication frequency with the closest child and cognitive function. *P < 0.05, **P < 0.01, ***P < 0.001.

To examine the interactions between intergenerational connections with the closest child and cognitive function, the study used a CLPM to model the interactions between intergenerational connections and cognitive function in 2011, 2013, 2015, and 2018 (see Figures 2A,B). Analyzing the individual paths in the model, as shown in Figure 2A, the frequency of meetings in 2013 had a negative predictive effect on cognitive function in 2015 (*β* = −0.040, *p* = 0.041), while the frequency of meetings in 2011 and 2015 did not have any significant predictive effect on cognitive function in 2013 and 2018, respectively (*β* = −0.035, 0.026, *p* = 0.092, 0.222). Cognitive function in all years was not significantly predictive of meeting frequency, respectively (*β* = −0.035, −0.022, 0.010, *p* = 0.092, 0.319, 0.651).

Analysis of the individual paths in the model, as shown in Figure 2B, revealed that communication frequency in 2011, 2013, and 2015 had a significant positive predictive effect on cognitive function in 2013, 2015, and 2018, respectively (*β* = 0.102, 0.068, 0.041, *p* < 0.001, *p* = 0.001, 0.045). Further, cognitive function in 2011, 2013, and 2015 had a significant positive predictive effect on communication frequency in 2013, 2015, and 2018, respectively. There was a significant positive predictive effect, respectively (*β* = 0.083, 0.083, 0.084, *p* = 0.001, 0.001, *p* < 0.001).

### Urban–rural and sex differences in the relationship between intergenerational connections with the closest child and cognitive function

Analyzing the urban–rural and sex differences in the relationship between intergenerational connections and cognitive function (see Supplementary Figures S1A–H), no significant predictive effect existed between meeting frequency and cognitive function among middle-aged and older adults in towns and cities in all years. Meeting frequency among middle-aged and older adults in the countryside in 2013 had a significant negative predictive effect on cognitive function in 2015 (*β* = −0.050, *p* = 0.042), and cognitive function among middle-aged and older adults in the countryside in 2011 had a significant negative predictive effect on meeting frequency in 2013 (*β* = −0.055, *p* = 0.039). No significant predictive effect existed between meeting frequency and cognitive function in the other years.

There was no significant predictive effect between meeting frequency and cognitive function in all years for male middle-aged and older adults. The meeting frequency for female in 2015 was a significant positive predictor of cognitive function in 2018 (*β* = 0.060, *p* = 0.047), and there was no significant predictive effect between meeting frequency and cognitive function for female in the other years.

Cognitive function among urban middle-aged and older adults in 2011 was a significant positive predictor of communication frequency in 2013 (*β* = 0.170, *p* = 0.020), and no significant predictive effect existed between communication frequency and cognitive function in the other years. Communication frequency among rural middle-aged and older adults in 2011, 2013, and 2015 was a significant positive predictor of cognitive function in 2013, 2015, and 2018, respectively (*β* = 0.116, 0.049, 0.073, *p* < 0.001, *p* = 0.048, 0.004). The cognitive function of rural middle-aged and older adults in 2011, 2013, and 2015 had a significant positive predictive effect on communication frequency in 2013, 2015, and 2018, respectively (*β* = 0.075, 0.084, 0.072, *p =* 0.017, 0.004, and 0.018).

Communication frequency for male in 2011, 2013, and 2015 was a significant positive predictor of cognitive function in 2013, 2015, and 2018, respectively (*β* = 0.103, 0.084, and 0.063, *p =* 0.001, 0.005, and 0.042). In 2013, cognitive function for male was a significant positive predictor of communication frequency in 2015 (*β* = 0.082, *p =* 0.011). There was no significant predictive effect between communication frequency and cognitive function in the remaining years for middle-aged and older male. Communication frequency in 2011 for female was a significant positive predictor of cognitive function in 2013 (*β* = 0.093, *p =* 0.001). Further, communication frequency in 2011 and 2015 for female was a significant positive predictor of cognitive function in 2013 and 2018 (*β* = 0.112, 0.092, *p =* 0.002, 0.005), and there was no significant predictive effect between communication frequency and cognitive function in the remaining years for female middle-aged and older adults.

### Mean of intergenerational connections with multiple children and cognitive function

#### Cross-sectional correlation and regression analysis between the mean of intergenerational connections with multiple children and cognitive function

Cross-sectional correlation analysis of intergenerational connections and cognitive function in 2011, 2013, 2015, and 2018 was conducted, and the results are shown in Table 3. It can be found that the correlation coefficients of frequency of meeting and cognitive function in 2011, 2013, 2015, and 2018 were 0.078, 0.063, 0.056, and 0.050, respectively, and all of them were significant at a 1‰ level of significance and indicated a significant positive correlation. The correlation coefficients of frequency of meeting and cognitive function in 2011, 2013, 2015, and 2018 and the correlation coefficients between frequency of communication and cognitive function were 0.261, 0.263, 0.282, and 0.242, respectively, and all were significant at 1% level of significance with significant positive correlation.

**Table 3:** Results of the correlation and regression analysis of the cross-sectional association between mean of intergenerational connections with multiple children and cognitive function.

Variables	Correlation coefficient	Regression coefficient
MeetingT1 – CognitiveT1 (*n* = 7168)	0.078***	0.142***
CommunicationT1 – CognitiveT1 (*n* = 7162)	0.261***	0.423***
MeetingT2 – CognitiveT2 (*n* = 7951)	0.063***	0.118***
CommunicationT2 – CognitiveT2 (*n* = 6398)	0.263***	0.491***
MeetingT3 – CognitiveT3 (*n* = 10027)	0.056***	0.108***
CommunicationT3 – CognitiveT3 (*n* = 8397)	0.282***	0.543***
MeetingT4 – CognitiveT4 (*n* = 9546)	0.050***	0.072***
CommunicationT4 – CognitiveT4 (*n* = 8032)	0.242***	0.341***

T1 represents the baseline, and T2, T3, and T4 represent the follow-up visits. ***P < 0.001.

Cross-sectional regression analysis of intergenerational connections and cognitive function in 2011, 2013, 2015, and 2018 were conducted. The results showed that frequency of meeting and frequency of communication were positive predictors of cognitive function in 2011, 2013, 2015, and 2018.

#### Longitudinal correlations between mean of intergenerational connections with multiple children and cognitive function

The longitudinal correlations between intergenerational connections and cognitive function in 2011, 2013, 2015, and 2018 were analyzed (see Supplementary Table S3). The frequency of meetings in 2013 was significantly positively correlated with cognitive functioning in 2018, and the correlation between the frequency of meetings and cognitive function at the other time points was not significant. The frequency of communication in the 4 time points over 8-year peorid was significantly positively correlated with cognitive function.

#### Cross-lagged panel analysis between the mean of intergenerational connections with multiple children and cognitive function

**Figure 3:**
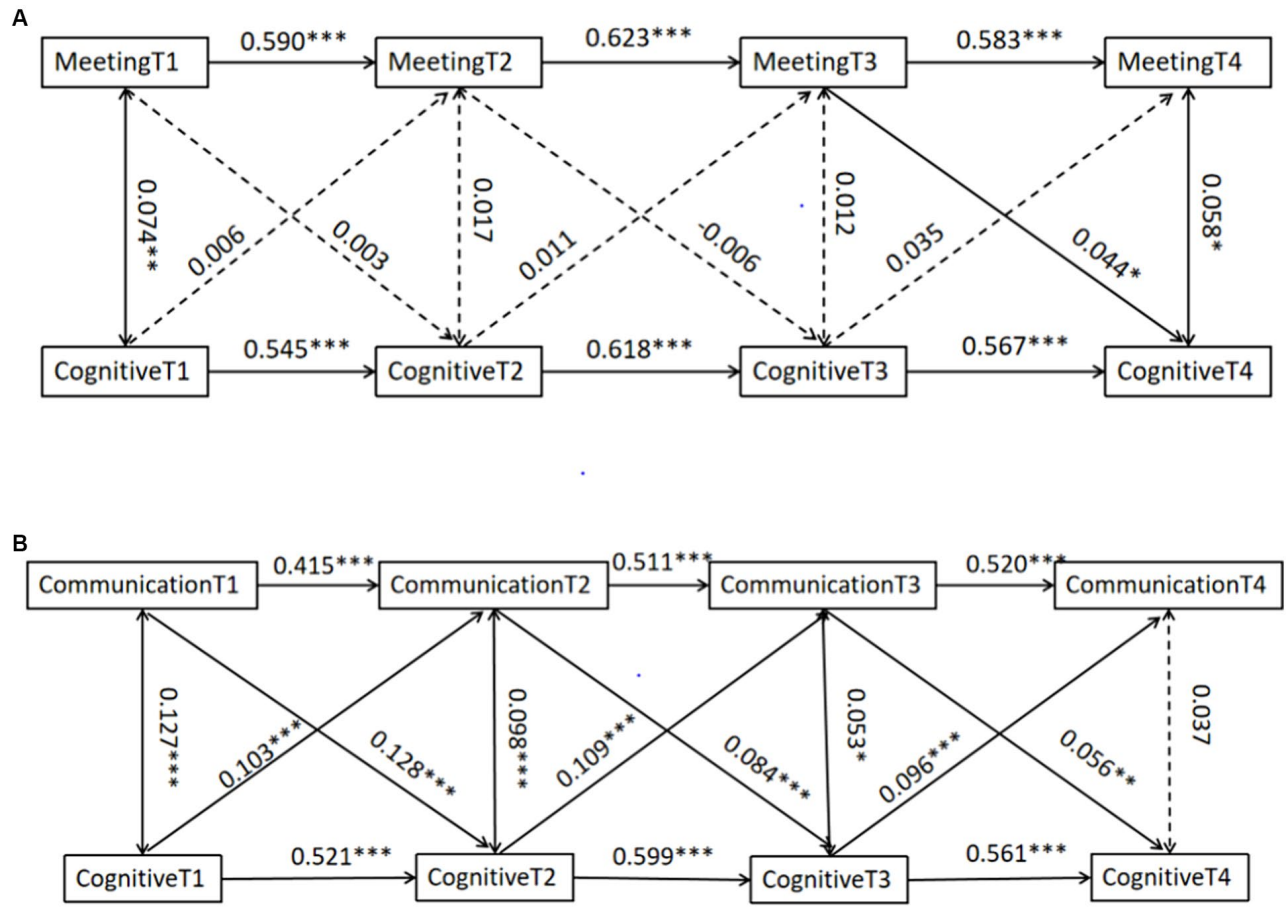
Cross-lagged panel model of the mean of intergenerational connections with multiple children and cognitive function. **(A)** Cross-lagged panel model of the mean of meeting frequency with multiple children and cognitive function. **(B)** Cross-lagged panel model of the mean of communication frequency with multiple children and cognitive function. *P < 0.05, **P < 0.01, ***P < 0.001.

To examine the interactions between the mean values of intergenerational connections with multiple children and cognitive function, the study used the CLPM to model the interactions between intergenerational connections and cognitive function in 2011, 2013, 2015, and 2018 (see Figures 3A,B). Analyzing the individual paths in the model in Figure 3A, we found that the frequency of meetings in 2015 had a positive predictive effect on cognitive function in 2018 (*β* = 0.044, *p* = 0.031), while the frequency of meetings in 2011 and 2013 did not have a significant predictive effect on the cognitive functioning in 2013 and 2015, respectively (*β* = 0.003, −0.006, *p* = 0.881, 0.750). The cognitive function of each year did not have a significant predictive effect on the frequency of meetings, respectively (*β* = 0.006, 0.011, 0.035, *p* = 0.778, 0.600, 0.118).

Analysis of the individual paths in the model of Figure 3B revealed that communication frequency in 2011, 2013, and 2015 was a significant positive predictor of cognitive function in 2013, 2015, and 2018, respectively (*β* = 0.128, 0.084, and 0.056, *p* < 0.001, 0.001, *p* = 0.008) and that cognitive function in 2011, 2013, and 2015 was a significant positive predictor of communication frequency in 2013, 2015, and 2018, respectively (*β* = 0.103, 0.109, 0.096, *p* < 0.001).

#### Urban–rural and sex differences in the relationship between the mean of intergenerational connections with multiple children and cognitive function

Analyzing the urban–rural and sex differences in the relationship between intergenerational connections and cognitive function (see Supplementary Figures S2A–H), the frequency of meeting among urban middle-aged and older adults in 2013 had a significant positive predictive effect on cognitive function in 2015 (*β* = 0.133, *p =* 0.045), and there was no significant predictive effect between the frequency of meeting and cognitive function in the other years. The meeting frequency among rural middle-aged and older adults in 2015 had a significant positive prediction effect on cognitive function in 2018 (*β* = 0.058, *p =* 0.024), and no significant predictive effect between meeting frequency and cognitive function in the other years among rural middle-aged and older adults.

There was no significant predictive effect between meeting frequency and cognitive function in all years for male middle-aged and older adults. Meeting frequency in 2015 for female was a significant positive predictor of cognitive function in 2018 (*β* = 0.081, *p =* 0.005), and there was no significant predictive effect between meeting frequency and cognitive function in the other years for female middle-aged and older adults.

Communication frequency of middle-aged and older adults in the townships in 2013 had a significant positive predictive effect on cognitive function in 2015 (*β* = 0.169, *p* = 0.009). The cognitive function of middle-aged and older adults in the townships in 2011 had a significant positive predictive effect on communication frequency in 2013 (*β* = 0.205, *p* = 0.005), while there was no significant predictive effect between communication frequency of middle-aged and older adults in the townships and cognitive function in the other years. The communication frequency of rural middle-aged and older adults in 2011, 2013, and 2015 had significant positive prediction effects on cognitive function in 2013, 2015, and 2018, respectively (*β* = 0.119, 0.054, 0.080, *p* < 0.001, *p* = 0.030, 0.002). The cognitive function of rural middle-aged and older adults in 2011, 2013, and 2015 had significant positive prediction effects on communication frequency in 2013, 2015, and 2018, respectively (*β* = 0.079, 0.101, 0.079, *p* = 0.01, 0.001, 0.007).

Communication frequency in 2011, 2013, and 2015 for male was a significant positive predictor of cognitive function in 2013, 2015, and 2018, respectively (*β* = 0.117, 0.082, and 0.071, *p* < 0.001, *p* = 0.006, and 0.023). Cognitive function in 2013 and 2015 for male was a significant positive predictor of communication frequency in 2015 and 2018, respectively (*β* = 0.095, 0.067, *p* = 0.002, 0.030). In 2011, male cognitive function was not a significant predictor of 2013 communication frequency (*β* = 0.018, *p* = 0.581). The 2011 and 2013 female communication frequency distributions were significant positive predictors of 2013 and 2015 cognitive function (*β* = 0.103, 0.059, *p* = 0.001, 0.042). The 2015 female communication frequency was not a significant predictor of 2018 cognitive function (*β* = 0.044, *p* = 0.143), while 2011, 2013, and 2015 female cognitive function was a significant positive predictor of 2013, 2015, and 2018 communication frequency, respectively (*β* = 0.148, 0.077, and 0.110, *p* < 0.001, *p =* 0.023, *p* < 0.001).

## Discussion

### Predictive relationships between intergenerational connections and cognitive function

#### Frequency of meeting with the closest child negatively predicts cognitive function

The results of cross-lagged panel analysis revealed that the frequency of meeting with the closest child in the first 2 years significantly negatively predicted cognitive function in the following 2 years, but cognitive function in the previous 2 years did not significantly predict the frequency of meeting with the closest child in the following 2 years. It is clear that the meeting frequency with the closest child has a negative effect on the cognitive function of middle-aged and older adults. This may be due to middle-aged and older adults providing more support to children with high intergenerational needs, such as those without a partner and having children those with health problems, and those with a lower level of education (29), which may be a burden to the older adults. Furthermore, from the perspective of middle-aged and older adults’ access to intergenerational support, the increase in the frequency of meeting with the closest child may be due to the mutual shirking of support obligations by the other children, especially when middle-aged and older adults suffer from health problems, such as disabilities, the children are chosen need provide more frequent support to their parents (30). Frequent meetings with the individual children may lead to intergenerational conflicts or ambivalece, which may exacerbate the anxiety and depression of the middle-aged and older adults (31) and further impair middle-aged and older adults’ cognitive function (32).

#### Frequency of communication with the closest child positively predicts cognitive function

This study found a significant positive predictive relationship between the frequency of communication with the closest child and cognitive function. A reciprocal positive effect between the frequency of communication with the closest child and cognitive function in middle-aged and older adults was observed. This is similar to previous findings (33, 34). Furthermore, the middle-aged and older adults may be more emotionally close to this child, thus providing a protective effect on cognitive functioning in middle-aged and older adults (35, 36). In addition, in rural areas, children who do not live with their parents provide more financial support to their parents than children who live with them (37). Children who work outside the home can earn higher financial rewards, and financial support has a positive impact on the physical and mental health of middle-aged and older adults (38).

The results of this study partly support the convoy model of social relations, in which communication can promote an individual’s physical and mental health, as well as the cognitive reserve theory, which suggests that a healthy lifestyle, involving interaction with adult children, is conducive to the development of cognitive function in middle-aged and older adults. However, the above theories and models emphasize their positive significance without focusing on their negative aspects. In this study, we found that factors such as the degree of harmony in intergenerational relationships may play an important role in influencing cognitive function. When intergenerational relationships are harmonious, intergenerational connections are conducive to the development of cognitive function, whereas when intergenerational relationships are prone to conflict, cognitive function tends to be impaired. To consider the bidirectional effects of intergenerational relationships, this study proposes the boat-carrying model, in which water can carry a boat or capsize it. In high-quality intergenerational relationships, various kinds of intergenerational support are conducive to the development of the physical and mental health of middle-aged and older adults, while in low-quality intergenerational relationships, the increase in various kinds of intergenerational support may harm the physical and mental health of middle-aged and older adults (36).

### Mean of intergenerational connections with multiple children positively predicts cognitive function

The results of the study showed that the mean value of intergenerational connections with multiple children, including frequency of meetings and frequency of communication, had a significant positive predictive relationship with cognitive function. It can be seen that there is a reciprocal positive effect between the mean value of intergenerational connections with multiple children and the cognitive function of middle-aged and older adults. This was consistent with the results of previous studies (34, 38, 39). While previous studies have shown that high-frequency and high-quality intergenerational connections are beneficial to the healthy longevity of middle-aged and older adults in terms of the frequency and depth of intergenerational connections, we also considered the effects of different ways of connecting, and the results showed that both forms of intergenerational connections with multiple children will protect cognitive function in middle-aged and older adults (40). High-quality intergenerational relationships are effective in improving the psychological well-being of middle-aged and older adults (31, 41, 42), thereby promoting cognitive function. The mean values of both the frequency of communication with the closest child and the frequency of communication with multiple children positively predicted cognitive functioning for eight consecutive years, suggesting that communication with children is a very important and necessary way of connecting with middle-aged and older adults.

Compared with the meeting frequency with the closest children, the average meeting frequency with multiple children had a positive impact on middle-aged and older adults, which suggested that children should provide appropriate care to their middle-aged and older parents and must not advocate for one child-bearing responsibility of intergenerational support; the burden should be balanced. Additionally, this study found that the cognitive function of middle-aged and older adults showed a decreasing trend with age, which suggested that we need to increase the attention we pay to cognitive aspects of middle-aged and older adults. While the meeting frequency with the closest child showed an increasing trend, the mean of meeting frequency with multiple children showed a decreasing trend. Further, the mean of communication frequency with multiple children showed an increasing trend, and the changes in the mean of meeting frequency with the closest child and meeting frequency with multiple children showed an opposite trend. Those results may suggest the intergenerational connections between middle-aged and older adults and their children gradually shifted from meeting to communicating, and intergenerational responsibilities gradually shifted to one child. This study proposes the theory of skewed intergenerational support, which suggests that as middle-aged and older adults grow older, the responsibility for intergenerational support is skewed toward the closest child, while this skewing leads to conflicts and friction between middle-aged and older adults and their closest child, which further affects cognitive function. In the process of interacting with their children, the favoritism of parents toward their closest children gradually increases over time (43). The closest children may see themselves as favored and feel obliged to bear most of the care burden, resulting in an uneven distribution of intergenerational support among children and gradually leaning toward the closest children, which aggravates the conflict within the family and further affects cognitive function. Perception of parental favoritism by other children leads to lower family intimacy, and when other children believe that their parents treat them fairly, intergenerational support is more evenly distributed within the family and family relationships are more harmonious, which benefits to the cognitive function in middle-aged and older adults (44). In Chinese, we have a saying “(yī wǎn shuǐ duān píng)”, which literally means “to hold a bowl of water level”. It’s used to emphasize the importance of treating everyone fairly and equally. And it is an important art in dealing with family parent-child relationships.

This study used a longitudinal design to examine the effects of the connections with the closest child and multiple children on cognitive function. It was found that different types of intergenerational connections showed varying mechanisms of action on cognitive function in middle-aged and older adults. This suggests that future intergenerational studies should not rely on averaged indicators from all children to represent the entire group. Instead, these studies should take into account individual differences among children within family.

### Urban–rural and sex differences in the impact of intergenerational connections on cognitive aging

In the context of urban–rural dual social structure, there are great differences in economic income, education level, medical security and infrastructure construction between urban and rural areas in China. which lead to differences in living conditions, lifestyle and family structure among the middle-aged and older adults in urban and rural areas (45–47). The study found that there were urban–rural differences in the effects of intergenerational connections on cognitive aging. For urban middle-aged and older adults, the average of the intergenerational connections with multiple children significantly positively predicted the cognitive function of urban middle-aged and older adults in the following 2 years, but none of the intergenerational connections with the closest child predicted cognitive function. For rural middle-aged and older adults, intergenerational connections, including the average of the closest child and multiple-children connections, significantly predicted the cognitive function of rural middle-aged and older adults in the following 2 years. One possible reason for this is that, in terms of the current aging model in China, rural middle-aged and older adults mainly rely on single-generation aging, and “bring up sons to support parents in their old age” is commonplace in the countryside (48). In cities and towns, the older adult care model is more diversified (49, 50), with the joint development of institutional care, community care, home care and so on. While rural middle-aged and older adults have a greater need for intergenerational support. Compared to their urban counterparts, middle-aged and older adults in rural areas engage in proportionally more intergenerational interactions within their overall interpersonal interactions. Furthermore, intergenerational connections, particularly with the closest child, have a more significant impact on cognitive function in rural middle-aged and older adults.

Existing studies show that there are sex differences in the development and decline of cognitive function (16, 17), bidirectional intergenerational support and emotional participation in middle-aged and older people (51). In most Chinese families, the relationship between their children and their mothers is obviously better than that between their children and their fathers. The study found that there were sex differences in the effect of intergenerational connections on cognitive aging, and in terms of male middle-aged and older adults, only frequency of communication, including with the closest child and the mean of multiple children, significantly and positively predicted cognitive function. Mother–child relationships were closer than father-child relationships (52), therefore, mothers are more likely than fathers to receive living arrangements and health support from their children (53). A closer and more harmonious intergenerational relationship can contribute to the maintenance and improvement of cognitive function.

The results of this study indicate that there are urban–rural and sex differences in the effects of intergenerational connections on cognitive function; additionally, the effects of intergenerational connections on cognitive function are more significant in rural female middle-aged and older adults, which suggests that we need to consider the effects of socio-demographic, psychological, and other factors in the mechanism between intergenerational connections and cognitive function (54).

### Study limitations and directions for further research

This study was limited to data availability and only involved the single factor intergenerational connections of intergenerational support. Future research can explore other intergenerational support methods, such as economic and housework support, between middle-aged and older parents and their children to comprehensively analyze the impact of intergenerational support on the physical and mental health of middle-aged and older adults. Financial support has an important impact on the physical and mental health of middle-aged and older adults. Whether it is diet, leisure, or medical treatment and health care, a certain amount of financial support is needed, and middle-aged and older parents’ access to household support can alleviate excessive pressure and reduce the likelihood of diseases (38). Concurrently, based on the theory of “role enhancement,” middle-aged and older adults who provide financial support and housework support obtain a sense of satisfaction and achievement in the process of helping their children, which promotes their mental health (55). Financial, housework, and emotional support are the three key points that improve the social pension system and the physical and mental health of middle-aged and the older adults. There is a close two-way intergenerational support between middle-aged and older parents and their children; future research can strengthen the comprehensive exploration of economic, housework, and emotional support. With the development of society, the parent–child relationship in China is also changing. Whether intergenerational relationships are changing alongside the aging model and socio-economic development requires further study.

CHARLS measures cognitive function assessment tools, derived from an adapted Chinese version of the Mini-Mental State Examination (MMSE), was mainly used to screen for cognitive function (56, 57). In the future, we could also use more accurate methods to measure cognitive function. Montreal Cognitive Assessment, Mini-Mental State Examination, and Clock Draw Test are also the most frequently studied objective screening tools (58). In addition, we can measure cognitive function using Clinical Dementia Rating (CDR), and Global Deterioration Scale (GDS), or some other functional checks methods (59).

When tracking and processing the data, it is worth exploring the effect of intergenerational connections with each child in a family in different years on the cognitive function of middle-aged and older adults, considering the intra-individual differences. However, current statistical methods are not yet able to support such statistical models. In the future, the use of nested data to combine the analysis of intra-individual differences and lagged causal effects should be considered.

## Data availability statement

The original contributions presented in the study are included in the article/Supplementary material, further inquiries can be directed to the corresponding author.

## Author contributions

SH: Data curation, Investigation, Methodology, Conceptualization, Formal analysis, Resources, Software, Writing – original draft. JG: Data curation, Supervision, Project administration, Validation, Writing – review & editing. MF: Conceptualization, Data curation, Formal analysis, Investigation, Software, Writing – review & editing. JY: Data curation, Investigation, Project administration, Validation, Writing – review & editing, Funding acquisition, Methodology, Supervision.

## References

[1] Jin W Zhang MY Sha Y Zhou JF. A heterogeneous study of impact of population ageing on structure and efficiency of service industry in Yangtze River Economic Belt. Res Environ Yangtze Basin. (2023) 32:1379–97.

[2] The National Bureau of Statistics of China. The seventh National Population Census Leading Group Office of the state council. The seventh National Population census bulletin~([1])(05)[N] China Information Daily (2021). 12(002) p.

[3] Fang EF Scheibye-Knudsen M Jahn HJ Li J Ling L Guo H. A research agenda for aging in China in the 21st century. Ageing Res Rev. (2015) 24:197–205. doi: 10.1016/j.arr.2015.08.003 26304837 PMC5179143

[4] Petersen RC. Mild cognitive impairment. Continuum. (2016) 22:404–18. doi: 10.1212/CON.0000000000000313, PMID: 27042901 PMC5390929

[5] Wu J Gu HM. Research progress on brain function network of amnestic mild cognitive impairment. J Clin Radiol. (2017) 36:1374–6. doi: 10.13437/j.cnki.jcr.2017.09.046

[6] Allen JS Bruss J Damasio H. The aging brain: the cognitive reserve hypothesis and hominid evolution. Am J Hum Biol. (2005) 17:673–89. doi: 10.1002/ajhb.20439 16254893

[7] Wang WH Huang MJ Lu ZJ Xie K-H Wang J-P Kou S. Influence of cognitive level on death risk in middle-aged and elderly people. Modern Prev Med. (2021) 48:3065–9.

[8] Kahn RL Antonucci T. Conboys over the life course: attachment roles and social support In: Life-span development and behavior, vol. 3 (1980). 253–86.

[9] Liu SS Ou YZ Wang HT. An overview of the research on social relations of the elderly: a convoy model perspective. Popul Dev. (2016) 22:90–7.

[10] Stern Y. Cognitive reserve and Alzheimer disease. Alzheimer Dis Assoc Disord. (2006) 20:S69–74. doi: 10.1097/00002093-200607001-00010 16917199

[11] Chan D Shafto M Kievit R Matthews F Sping M Valenzuela M. Lifestyle activities in mid-life contribute to cognitive reserve in late-life, independent of education, occupation, and late-life activities. Neurobiol Aging. (2018) 70:180–3. doi: 10.1016/j.neurobiolaging.2018.06.012, PMID: 30025291 PMC6805221

[12] Oosterhuis EJ Slade K May PJC Nuttall HE. Toward an understanding of healthy cognitive aging: the importance of lifestyle in cognitive reserve and the scaffolding theory of aging and cognition. J Gerontol Series B. (2023) 78:777–88. doi: 10.1093/geronb/gbac197, PMID: 36546399 PMC10174283

[13] Tang C. A review of modernization theory and its development on family. Soc Stud. (2010) 25:199–222.

[14] Abrams D Crisp R Marques S Fagg E Bedford L Provias D. Threat inoculation: experienced and imagined intergenerational contact prevents stereotype threat effects on older people's math performance. Psychol Aging. (2008) 23:934–9. doi: 10.1037/a0014293, PMID: 19140662

[15] Ran XX Hu HW. Urban-rural disparity, digital divide and health inequality of the elderly. Popul J. (2022) 44:46–58. doi: 10.16405/j.cnki.1004-129X.2022.03.004

[16] Tomioka K Kurumatani N Hosoi H. Social participation and cognitive decline among community-dwelling older adults: a community-based longitudinal study. J Gerontol Series B. (2018) 73:799–806. doi: 10.1093/geronb/gbw059, PMID: 27194753

[17] Zhu Y Chen Y Crimmins EM Zissimopoulos JM. Sex, race, and age differences in prevalence of dementia in Medicare claims and survey data. J Gerontol Series B. (2021) 76:596–606. doi: 10.1093/geronb/gbaa083, PMID: 32588052 PMC7887731

[18] Wang P Li SZ. Determinants of cognitive function of the rural elderly in China. J Air Force Med Univ. (2007) 17:1621–3.

[19] Gong J Wang G Wang Y Chen X Chen Y Meng Q. Nowcasting and forecasting the care needs of the older population in China: analysis of data from the China health and retirement longitudinal study (CHARLS). Lancet Public Health. (2022) 7:e1005–13. doi: 10.1016/S2468-2667(22)00203-1, PMID: 36423656 PMC9741660

[20] Liu Y Wang J Yan Z Huang R Cao Y Song H. Impact of child’s migration on health status and health care utilization of older parents with chronic diseases left behind in China. BMC Public Health. (2021) 21:1892–9. doi: 10.1186/s12889-021-11927-x, PMID: 34666723 PMC8527753

[21] Wang S Chen R Liu Q Zhan S Li L. Comprehensive treatment of hypertension middle-aged and elderly people: cross-sectional survey data from the China health and retirement longitudinal study (CHARLS). Lancet. (2015) 386:S67. doi: 10.1016/S0140-6736(15)00648-0

[22] Zhao Y Atun R Oldenburg B McPake B Tang S Mercer SW. Physical multimorbidity, health service use, and catastrophic health expenditure by socioeconomic groups in China: an analysis of population-based panel data. Lancet Glob Health. (2020) 8:e840–9. doi: 10.1016/S2214-109X(20)30127-3, PMID: 32446349 PMC7241981

[23] Liu Y Gao X Zhang Y Zeng M Liu Y Wu Y. Geographical variation in dementia prevalence across China: a geospatial analysis. Lancet Reg Health West Pac. (2024) 47:101117. doi: 10.1016/j.lanwpc.2024.101117, PMID: 38974661 PMC11225804

[24] Razani J Wong JT Dafaeeboini N Edwards-Lee T Lu P Alessi C. Predicting everyday functional abilities of dementia patients with the Mini-mental state examination. J Geriatr Psychiatry Neurol. (2009) 22:62–70. doi: 10.1177/0891988708328217, PMID: 19196632 PMC2679691

[25] Cole DA Maxwell SE. Testing mediational models with longitudinal data: questions and tips in the use of structural equation modeling. J Abnorm Psychol. (2003) 112:558–77. doi: 10.1037/0021-843X.112.4.558, PMID: 14674869

[26] Fang JY Wen ZL Huang GM. Exploring the longitudinal relations: based on longitudinal models with cross-lagged structure. J Psychol Sci. (2023) 46:734–41. doi: 10.16719/j.cnki.1671-6981.20230328

[27] Hamaker EL Kuiper RM Grasman RP. A critique of the cross-lagged panel model. Psychol Methods. (2015) 20:102–16. doi: 10.1037/a0038889 25822208

[28] Berry D Willoughby MT. On the practical interpretability of cross-lagged panel models: rethinking a developmental workhorse. Child Dev. (2017) 88:1186–206. doi: 10.1111/cdev.12660, PMID: 27878996

[29] Kalmijn M. How mothers allocate support among adult children: evidence from a multiactor survey. J Gerontol Series B. (2013) 68:268–77. doi: 10.1093/geronb/gbs110, PMID: 23231834

[30] Cheng YP Birditt KS Zarit SH Fingerman KL. Young adults’ provision of support to middle-aged parents. J Gerontol B Psychol Sci Soc Sci. (2015) 70:407–16. doi: 10.1093/geronb/gbt108, PMID: 24162441 PMC4542646

[31] Zhou A Song Y Li X Hu B Chen Y Cui P. Functional limitation and happiness among older adults: the multiple mediating role of intergenerational support and intergenerational relationship. Front Public Health. (2023) 11:216. doi: 10.3389/fpubh.2023.1249216, PMID: 37905237 PMC10613474

[32] Foong HF Hamid TA Ibrahim R Haron SA. Moderating effect of intrinsic religiosity on the relationship between depression and cognitive function among community-dwelling older adults. Aging Ment Health. (2018) 22:483–8. doi: 10.1080/13607863.2016.1274376, PMID: 28060527

[33] Hwang W Fu X Brown MT Silverstein M. Digital and non-digital solidarity between older parents and their middle-aged children: associations with mental health during the COVID-19 pandemic. Int J Environ Res Public Health. (2022) 19:12560. doi: 10.3390/ijerph191912560, PMID: 36231855 PMC9566078

[34] Wang P Li SZ Zhang WJ. The effects of intergenerational supports on the cognitive function of the rural elderly in China. Psychol Sci. (2005) 6:222–5.

[35] Ikeda A Iso H Kawachi I Yamagishi K Inoue M Tsugane S. Living arrangement and coronary heart disease: the JPHC study. Heart. (2008) 95:577–83. doi: 10.1136/hrt.2008.149575, PMID: 19066191

[36] Zheng R Yu M Huang L Wang F Gao B Fu D. Effect of intergenerational exchange patterns and intergenerational relationship quality on depressive symptoms in the elderly: an empirical study on CHARLS data. Front Public Health. (2022) 10:1009781. doi: 10.3389/fpubh.2022.1009781, PMID: 36262237 PMC9574018

[37] Zimmer Z Kwong J. Family size and support of older adults in urban and rural China: current effects and future implications. Demography. (2003) 40:23–44. doi: 10.1353/dem.2003.0010, PMID: 12647512

[38] Mei XW Feng X. Intergenerational support and health of the rural elderly——based on families of returning migrant workers. Popul Dev. (2023) 29:122–37.

[39] Li CH Wu WC. Generational interaction and elderly mortality risk. Popul J. (2017) 39:78–87. doi: 10.16405/j.cnki.1004-129X.2017.03.007

[40] Bengtson V Giarrusso R Mabry JB Silverstein M. Solidarity, conflict, and ambivalence: complementary or competing perspectives on intergenerational relationships? J Marriage Fam. (2002) 64:568–76. doi: 10.1111/j.1741-3737.2002.00568.x

[41] Shen C Wang DN Gao XX Zhao R Dong C Gu ZF. A study on the impact of the number of family generations on intergenerational support for centenarians: a study in a Chinese ‘longevity city’. Psychogeriatrics. (2023) 23:908–17. doi: 10.1111/psyg.13009, PMID: 37652078

[42] Yan X Wu W Chen X Xu G Yu S Li S. Intergenerational caregiving on mental health of middle-aged and older adults in China: empirical insights. Front Public Health. (2023) 11:4062. doi: 10.3389/fpubh.2023.1224062, PMID: 37483932 PMC10358982

[43] Jill Suitor J Gilligan M Pillemer K. Continuity and change in mothers' favoritism toward offspring in adulthood. J Marriage Fam. (2013) 75:1229–47. doi: 10.1111/jomf.12067, PMID: 38689711 PMC11060705

[44] Suitor JJ Sechrist J Plikuhn M Pardo ST Gilligan M Pillemer K. The role of perceived maternal favoritism in sibling relations in midlife. J Marriage Fam. (2009) 71:1026–38. doi: 10.1111/j.1741-3737.2009.00650.x, PMID: 20104251 PMC2810864

[45] Ma C Song Z Zong Q. Urban-rural inequality of opportunity in health care: evidence from China. Int J Environ Res Public Health. (2021) 18:7792. doi: 10.3390/ijerph18157792 34360084 PMC8345655

[46] Wang X Hai S Cai P. Urban–rural disparity of child poverty in China: spatio-temporal changes and influencing factors. J Rural Stud. (2022) 91:170–83. doi: 10.1016/j.jrurstud.2022.03.005

[47] Zhang J Jin S Torero M Li T. Teachers and urban-rural gaps in educational outcomes. Am J Agric Econ. (2018) 100:1207–23. doi: 10.1093/ajae/aay009

[48] Zhang D Zheng L Chu SZ. “Raising sons for old age” or “raising daughters for old age”?--an empirical analysis of influences of children scale and gender structure on family intergenerational support. Popul Dev. (2021) 27:96–109.

[49] Chen XY Jiang JC. Evaluation and comparison of Wuhan pension model based on cost-benefit method. Hubei Soc Sci. (2020) 9:43–54. doi: 10.13660/j.cnki.42-1112/c.015457

[50] Liu Y Li XL. Research on the comparison and optimization path of community intelligent pension model in China in the digital era. E-Gov. (2022) 5:112–24. doi: 10.16582/j.cnki.dzzw.2022.05.011

[51] Irshad CV Behera DK Dash U. Participation of older adults in the intra-household decision-making activities: evidence from the longitudinal ageing study in India. J Adult Prot. (2021) 23:325–36. doi: 10.1108/JAP-03-2021-0013/full/html

[52] Van Houdt K Kalmijn M Ivanova K. Perceptions of closeness in adult parent–child dyads: asymmetry in the context of family complexity. J Gerontol Series B. (2020) 75:2219–29. doi: 10.1093/geronb/gbaa122, PMID: 32777051 PMC7751165

[53] Quashie NT Andrade FCD Meltzer G García C. Living arrangements and intergenerational support in Puerto Rico: are fathers disadvantaged? J Gerontol B Psychol Sci Soc Sci. (2022) 77:2078–90. doi: 10.1093/geronb/gbac044 35240683 PMC9683498

[54] Xiao F Cao S Xiao MZ Xie LL Zhao QH. Patterns of home care and community support preferences among older adults with disabilities in China: a latent class analysis. BMC Geriatr. (2023) 23:117. doi: 10.1186/s12877-023-03830-4, PMID: 36869322 PMC9983178

[55] Wang P Zhang WJ Wang J. Impact of the intergenerational family support on the mental health of the rural elderly. Chin J Gerontol. (2017) 37:4893–6.

[56] Ma C Li M Wu C. Cognitive function trajectories and factors among Chinese older adults with subjective memory decline: CHARLS longitudinal study results (2011–2018). Int J Environ Res Public Health. (2022) 19:16707. doi: 10.3390/ijerph192416707, PMID: 36554588 PMC9778675

[57] Xiang Y Zare H Guan C Gaskin D. The impact of rural-urban community settings on cognitive decline: results from a nationally-representative sample of seniors in China. BMC Geriatr. (2018) 18:323. doi: 10.1186/s12877-018-1003-0, PMID: 30594142 PMC6311043

[58] Elie IG Helen H Henry L. A review of cognitive screening tools in cancer. Curr Opin Support Palliat Care. (2017) 11:24–31. doi: 10.1097/SPC.0000000000000257 28009651

[59] Jeon DW Ju HB Jung DU Kim SJ Shim JC Moon JJ. Usefulness of the University of California san Diego Performance-based skills assessment for the evaluation of cognitive function and activities of daily living function in patients with cognitive impairment. Aging Ment Health. (2019) 23:46–52. doi: 10.1080/13607863.2017.1393796, PMID: 29068696

