# Supplementary material for: Role of intergenerational connections in cognitive aging: Evidence from a Chinese longitudinal study
Source: Front Public Health. 2024 Aug 21;12:1396620. doi: 10.3389/fpubh.2024.1396620 (PMC11371578; doi:10.3389/fpubh.2024.1396620)
Supplement: Supplementary file 1 [file Presentation_1.pdf]

Supplementary Material

Supplementary Table 1 Sample Descriptive Statistical Results (n=1,480)

Variables	2011	2013	2015	2018	F/ C^2
Controlled variables:					
Sex:					-
Male	767(51.8)	767(51.8)	767(51.8)	767(51.8)	
Female	713(48.2)	713(48.2)	713(48.2)	713(48.2)	
Age	62.56±8.57	64.55±8.56	66.55±8.56	9.53±8.58	-
Residence:					-
urban	417(28.2)	417(28.2)	251(17.0)	257(17.4)	
Rural	1,063(71.8)	1,063(71.8)	1,224(83.0)	1,223(82.6)	
Wage income:					C^2=12.066**
Have wage income	131(8.9)	179(12.1)	184(12.5)	175(11.8)	
No wage income	1,347(91.1)	1,299(87.9)	1,294(87.6)	1,305(88.2)	
Marital status:					C^2=23.283***
Married and living with a spouse	921(62.2)	875(59.1)	845(57.1)	795(53.7)	
Other	559(37.8)	605(40.9)	635(42.9)	685(46.3)	
Education level:					-
Primary school education or above	370(25.0)	110(19.1)	163(21.0)	357(24.1)	
Primary school education or below	1,110(75.0)	467(80.9)	613(79.0)	1,123(75.9)	
Chronic disease condition:					C^2=75.180***

Suffering from chronic disease	1,061(72.2)	1,155(79.1)	1,203(85.4)	675(79.6)	
No chronic disease	408(27.8)	306(20.9)	206(14.6)	173(20.4)	
Depression	9.69±6.73	8.80±6.13	9.06±6.75	9.48±6.82	F=5.460***
Dependent variable: cognitive function					
	9.98±4.05	10.09±4.09	9.55±4.12	6.31±3.12	F=319.880***
Independent variables: intergenerational connection (multiple children at the maximum value)					
Meeting frequency	6.78±2.75	7.03±2.67	7.13±2.59	6.78±2.55	F=6.558***
Communication frequency	7.06±2.43	7.05±2.26	7.11±2.25	7.24±2.31	F=2.175
Intergenerational connections (multiple children on average)					
Meeting frequency	5.45±2.07	5.42±1.82	5.52±1.84	5.26±1.77	F=5.012**
Communication frequency	6.11±2.37	6.42±2.25	6.41±2.20	6.57±2.30	F=10.687***

Note: The data are M ± SD or n (%), * P <0.05, ** P <0.01, *** P <0.001, the same below.

Supplementary Table 2 Results of the longitudinal correlation between intergenerational connection with the closest child and cognitive function (n=1,480)

Variables	MeetingT1	MeetingT2	MeetingT3	MeetingT4	CommunicationT1	CommunicationT2	CommunicationT3	CommunicationT4	CognitiveT1	CognitiveT2	CognitiveT3	CognitiveT4
MeetingT1	1.000											
MeetingT2	0.622***	1.000										
MeetingT3	0.561***	0.602***	1.000									
MeetingT4	0.526***	0.552***	0.572***	1.000								
CommunicationT1	0.043	0.018	0.019	-0.020	1.000							
CommunicationT2	-0.068**	-0.001	-0.040	-0.004	0.387***	1.000						
CommunicationT3	-0.108***	-0.054*	-0.033	-0.057*	0.369***	0.517***	1.000					
CommunicationT4	-0.098***	-0.084**	-0.066*	-0.041	0.333***	0.465***	0.515***	1.000				
CognitiveT1	-0.021	-0.043	-0.014	-0.032	0.150***	0.131***	0.142***	0.166***	1.000			
CognitiveT2	-0.057*	-0.039	-0.044	-0.060*	0.182***	0.157***	0.154***	0.199***	0.589***	1.000		
CognitiveT3	-0.055*	-0.062*	-0.056*	-0.081**	0.196***	0.169***	0.165***	0.191***	0.578***	0.654***	1.000	
CognitiveT4	-0.048	-0.025	-0.029	-0.037	0.163***	0.132***	0.141***	0.153***	0.570***	0.609***	0.628***	1.000

Note: *P<0.05;**P<0.01;***P<0.001.

Supplementary Table 3 Results of the longitudinal correlation between mean of intergenerational connections with multiple children and cognitive function (n=1,480)

Variables	MeetingT1	MeetingT2	MeetingT3	MeetingT4	CommunicationT1	CommunicationT2	CommunicationT3	CommunicationT4	CognitiveT1	CognitiveT2	CognitiveT3	CognitiveT4

MeetingT1	1.000											
MeetingT2	0.591***	1.000										
MeetingT3	0.561***	0.622***	1.000									
MeetingT4	0.497***	0.540***	0.601***	1.000								
CommunicationT1	-0.051*	-0.082**	-0.075**	-0.103***	1.000							
CommunicationT2	-0.001	0.109***	0.008	0.027	0.434***	1.000						
CommunicationT3	-0.033	-0.01	0.074**	-0.008	0.413***	0.535***	1.000					
CommunicationT4	-0.018	-0.032	-0.018	0.064*	0.376***	0.501***	0.567***	1.000				
CognitiveT1	0.031	0.026	0.035	0.038	0.186***	0.175***	0.187***	0.204***	1.000			
CognitiveT2	0.010	0.022	0.025	0.016	0.231***	0.219***	0.210***	0.232***	0.589***	1.000		
CognitiveT3	0.013	0.011	0.021	-0.000	0.254***	0.223***	0.209***	0.234***	0.578***	0.654***	1.000	
CognitiveT4	0.015	0.058*	0.042	0.039	0.209***	0.180***	0.186***	0.200***	0.570***	0.609***	0.628***	1.000

Note: *P<0.05;**P<0.01;***P<0.001.

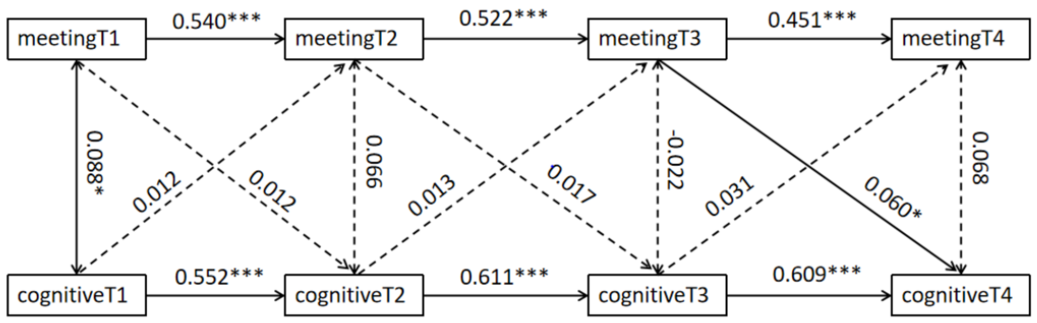

(A)

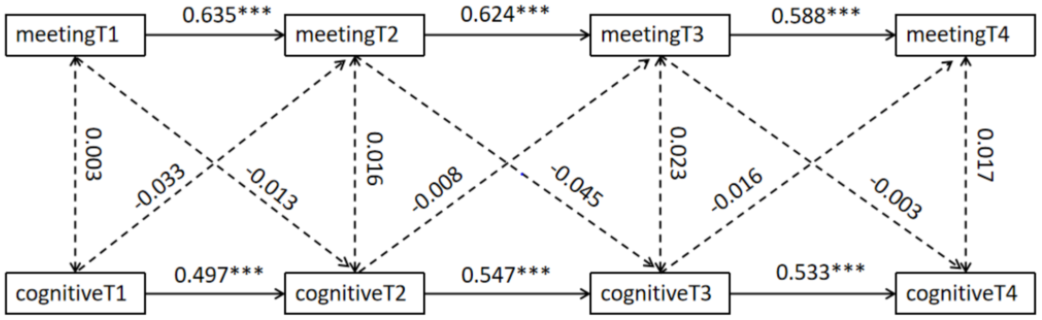

(B)

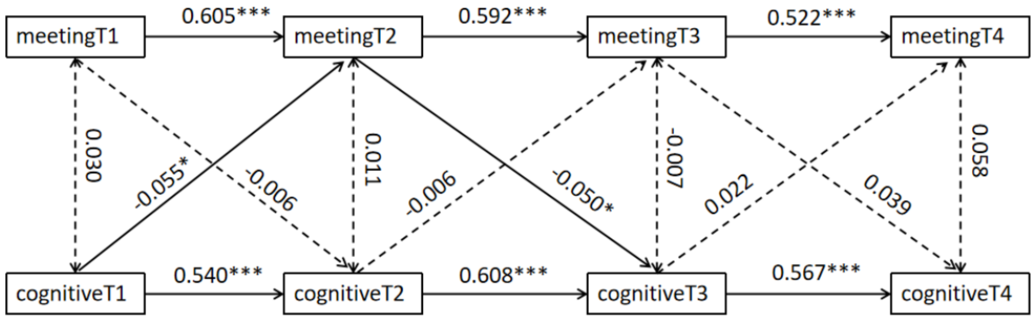
meetingT1
0.605***
meetingT2
0.592***
meetingT3
0.522***
meetingT4
0.030
-0.055*
-0.006
0.011
-0.006
-0.050*
-0.007
0.022
0.039
0.058
cognitiveT1
0.540***
cognitiveT2
0.608***
cognitiveT3
0.567***
cognitiveT4

(C)

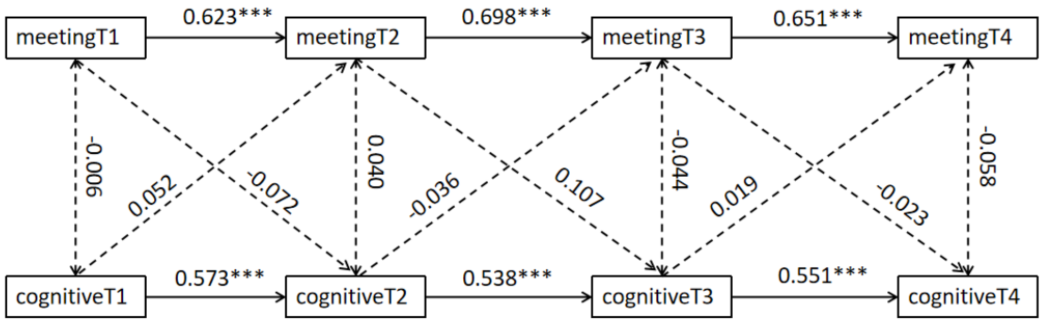
meetingT1
0.623***
meetingT2
0.698***
meetingT3
0.651***
meetingT4
-0.006
0.052
-0.072
0.040
-0.036
0.107
-0.044
0.019
-0.023
-0.058
cognitiveT1
0.573***
cognitiveT2
0.538***
cognitiveT3
0.551***
cognitiveT4

(D)

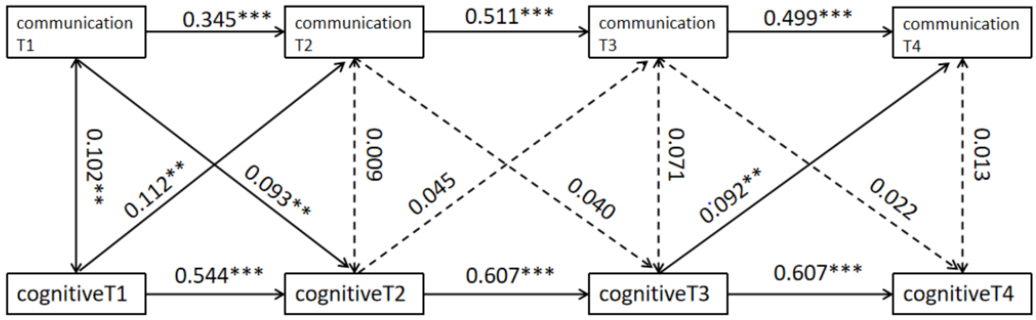
communication T1
0.345***
communication T2
0.511***
communication T3
0.499***
communication T4
0.102**
0.112**
0.093**
0.009
0.045
0.040
0.071
0.092**
0.022
0.013
cognitiveT1
0.544***
cognitiveT2
0.607***
cognitiveT3
0.607***
cognitiveT4

(E)

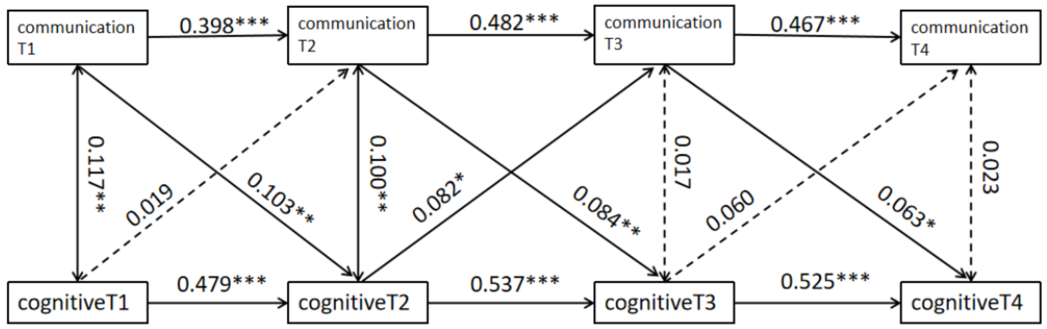

(F)

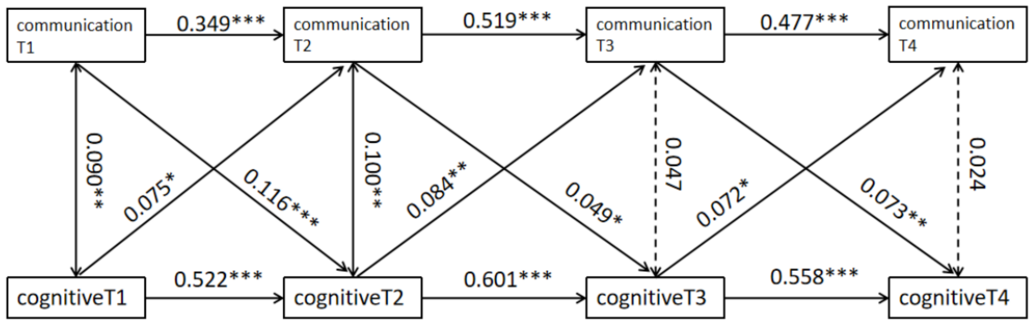

(G)

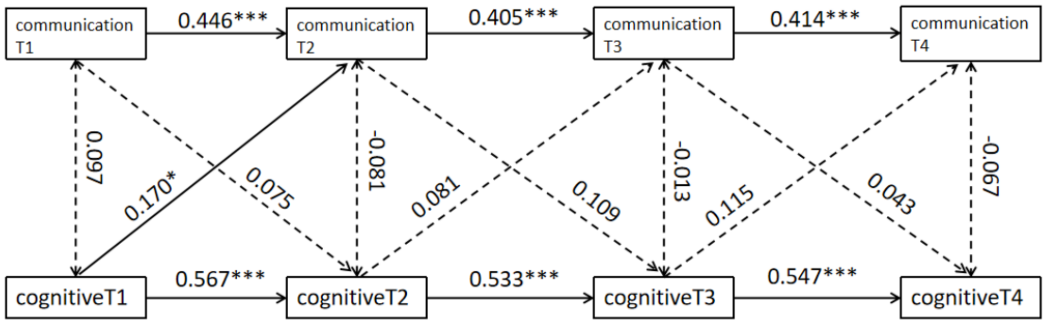

(H)

Supplementary Figures 1 Urban-rural and Gender Differences in the Relationship Between Intergenerational Connection with the Closest Child and Cognitive Function (Note: Figures 1(A) for female, (B) for male, (C) for rural, (D) for urban, (E) for female, (F) for male, (G) for rural and (H) for urban. *P<0.05;**P<0.01;***P<0.001)

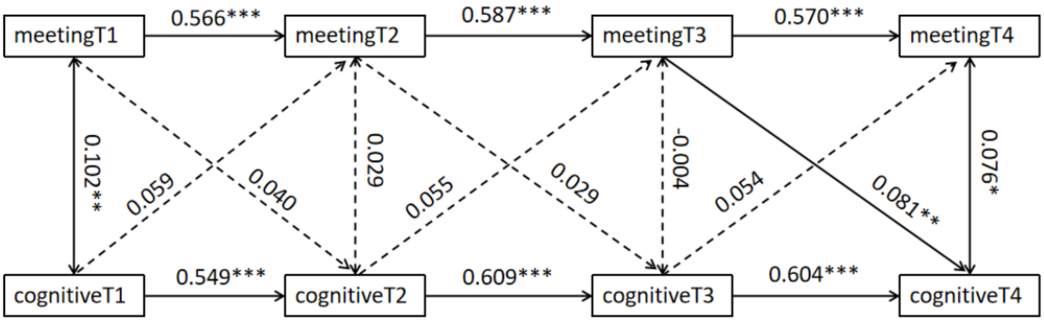
meetingT1
meetingT2
meetingT3
meetingT4
cognitiveT1
cognitiveT2
cognitiveT3
cognitiveT4
0.566***
0.587***
0.570***
0.549***
0.609***
0.604***
0.102**
0.059
0.040
0.029
0.055
0.029
-0.004
0.054
0.081**
0.076*

(A)

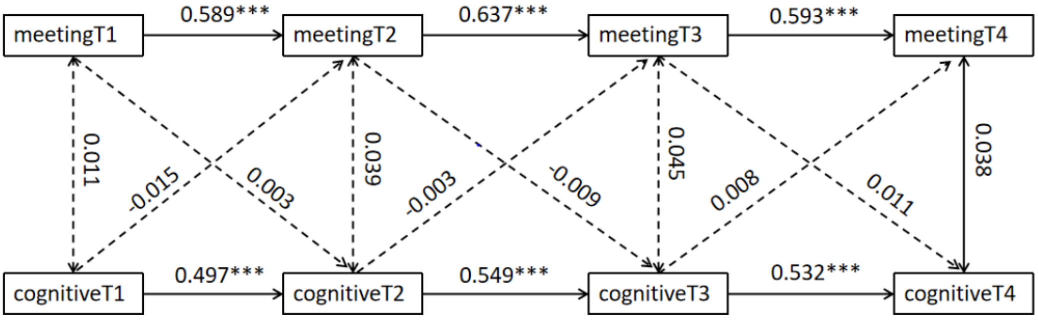
meetingT1
meetingT2
meetingT3
meetingT4
cognitiveT1
cognitiveT2
cognitiveT3
cognitiveT4
0.589***
0.637***
0.593***
0.497***
0.549***
0.532***
0.011
-0.015
0.003
0.039
-0.003
-0.009
0.045
0.008
0.011
0.038

(B)

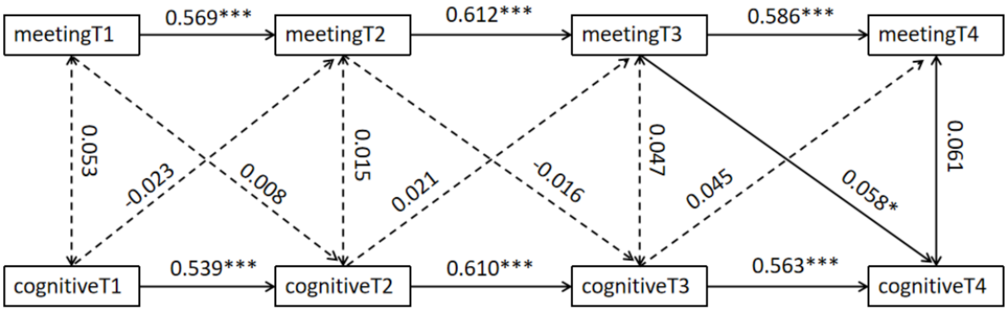
meetingT1
meetingT2
meetingT3
meetingT4
cognitiveT1
cognitiveT2
cognitiveT3
cognitiveT4
0.569***
0.612***
0.586***
0.539***
0.610***
0.563***
0.053
-0.023
0.008
0.015
0.021
-0.016
0.047
0.045
0.058*
0.061

(C)

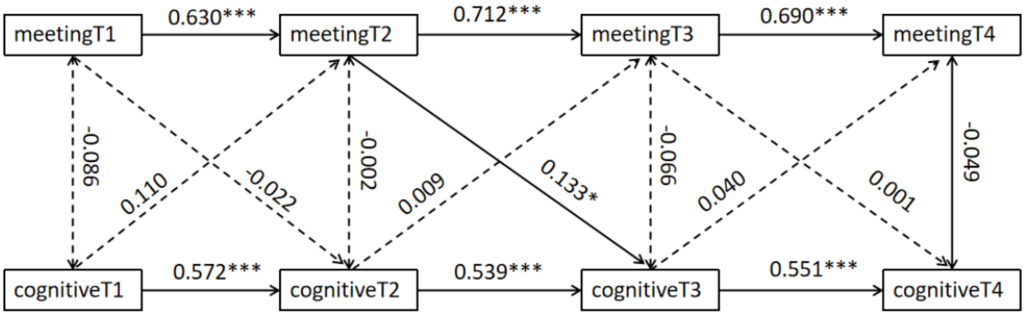
meetingT1
meetingT2
meetingT3
meetingT4
cognitiveT1
cognitiveT2
cognitiveT3
cognitiveT4
0.630***
0.712***
0.690***
0.572***
0.539***
0.551***
-0.086
0.110
-0.022
-0.002
0.009
0.133*
-0.066
0.040
0.001
-0.049

(D)

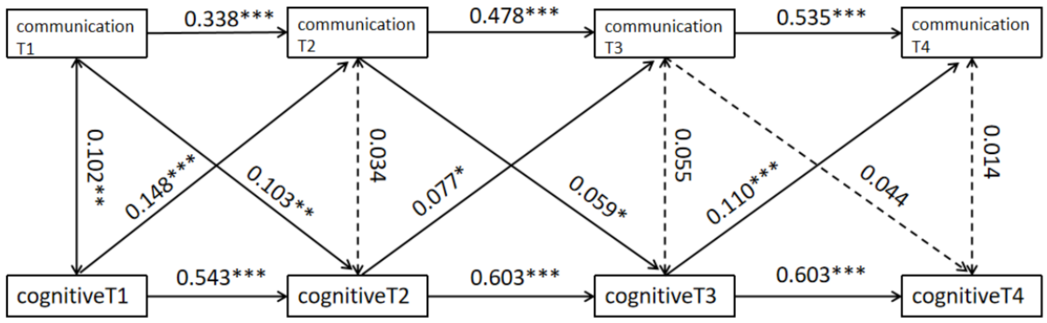
communication T1
0.338***
communication T2
0.478***
communication T3
0.535***
communication T4
0.102**
0.148***
0.103**
0.034
0.077*
0.059*
0.055
0.110***
0.044
0.014
cognitiveT1
0.543***
cognitiveT2
0.603***
cognitiveT3
0.603***
cognitiveT4

(E)

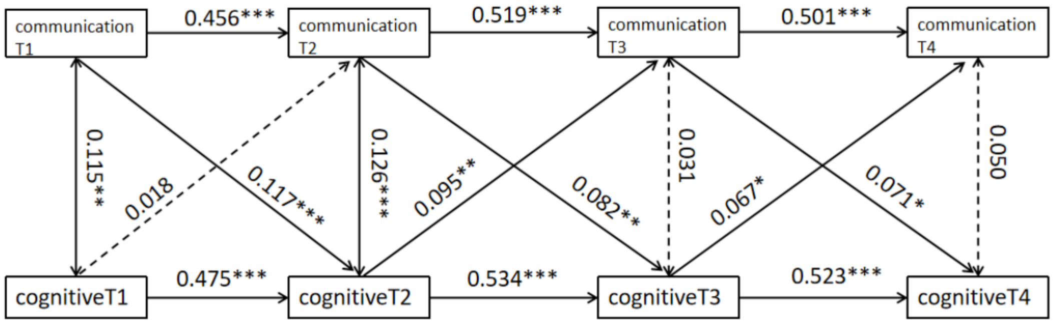
communication T1
0.456***
communication T2
0.519***
communication T3
0.501***
communication T4
0.115**
0.018
0.117***
0.126***
0.095**
0.082**
0.031
0.067*
0.071*
0.050
cognitiveT1
0.475***
cognitiveT2
0.534***
cognitiveT3
0.523***
cognitiveT4

(F)

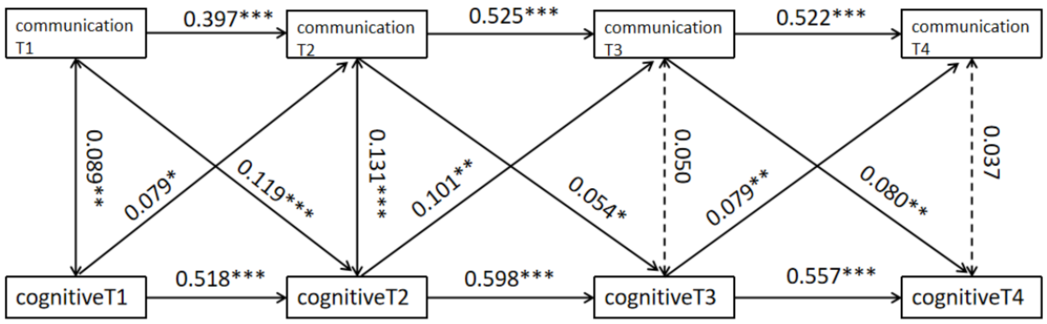
communication T1
0.397***
communication T2
0.525***
communication T3
0.522***
communication T4
0.089**
0.079*
0.119***
0.131***
0.101**
0.054*
0.050
0.079**
0.080**
0.037
cognitiveT1
0.518***
cognitiveT2
0.598***
cognitiveT3
0.557***
cognitiveT4

(G)

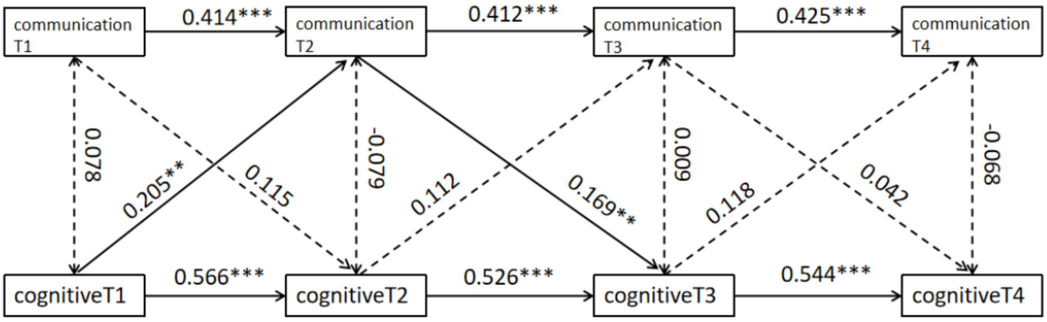

(H)

Supplementary Figures 2 Urban-rural and Gender Differences in the Relationship Between the Mean of Intergenerational Connections with Multiple Children and Cognitive Function (Note: Figures 2(A) for female, (B) for male, (C) for rural, (D) for urban, (E) for female, (F) for male, (G) for rural and (H) for urban. *P<0.05;**P<0.01;***P<0.001)
